# Efficacy and Safety of Novel Aspirin Formulations: A Randomized, Double-Blind, Placebo-Controlled Study

**DOI:** 10.3390/pharmaceutics14010187

**Published:** 2022-01-13

**Authors:** Rocco Mollace, Micaela Gliozzi, Roberta Macrì, Annamaria Tavernese, Vincenzo Musolino, Cristina Carresi, Jessica Maiuolo, Carolina Muscoli, Carlo Tomino, Giuseppe Maria Rosano, Massimo Fini, Maurizio Volterrani, Bruno Silvestrini, Vincenzo Mollace

**Affiliations:** 1 Department of Health Science, Institute of Research for Food Safety & Health IRC-FSH, University Magna Graecia, 88100 Catanzaro, Italy; rocco.mollace@gmail.com (R.M.); micaela.gliozzi@gmail.com (M.G.); robertamacri85@gmail.com (R.M.); an.tavernese@gmail.com (A.T.); xabaras3@hotmail.com (V.M.); carresi@unicz.it (C.C.); jessicamaiuolo@virgilio.it (J.M.); muscoli@unicz.it (C.M.); bruno.silvestrini@alice.it (B.S.); 2 IRCCS San Raffaele Pisana, Via di Valcannuta, 00163 Rome, Italy; carlo.tomino@uniroma5.it (C.T.); giuseppe.rosano@sanraffaele.it (G.M.R.); massimo.fini@sanraffaele.it (M.F.); maurizio.volterrani@sanraffaele.it (M.V.)

**Keywords:** aspirin, coronary artery disease prevention, gastric protection, micronization, collagen cogrinding

## Abstract

Low-dose aspirin represents the best option in the secondary prevention of coronary artery disease, but its extensive use in primary prevention is limited by the occurrence of gastric mucosal lesions and increased risk of bleeding. We investigated the safety profile of a novel sublingual aspirin formulation in 200 healthy volunteers, randomly assigned to ten (*n* = 20 each) different 7-day once-daily treatment regimens. Gastric mucosal injury based on the modified Lanza score (MLS), the histopathology of gastric mucosa and the serum determination of thromboxane B_2_ (TXB_2_) and urinary 11-dehydro-TXB_2_ levels were evaluated at basal as well as after 7 days of each placebo or aspirin treatment regimen. In Groups A and B (placebo—oral and sublingual, respectively), no changes in MLS and in gastric mucosal micro-vessel diameter were found at day 7. In contrast, in Groups C and D (oral standard aspirin—100 and 50 mg daily, respectively), the median MLS was significantly increased. Very few changes were found in Groups E and F (standard sublingual aspirin—100 and 50 mg, respectively). Groups G and H (oral administration of micronized collagen-cogrinded aspirin) showed gastric protection compared to Groups C and D. Moreover, Groups I and L (sublingual collagen-cogrinded aspirin—100 and 50 mg, respectively) showed a significant reduction (Group I) or total abolition (Group L) of gastric mucosal lesions and no difference compared to the standard one in serum TXB_2_ and urinary 11-dehydro-TXB_2_ levels. In conclusion, our data show that the new formulation leads to a better safety profile compared to standard aspirin, representing a better therapeutic option for extended use in primary and secondary prevention of cardiovascular diseases.

## 1. Introduction

Aspirin is the most successful drug in history. It was discovered over a hundred years ago, and, now, one billion tablets are consumed every year worldwide. In particular, evidence has been collected showing that low-dose aspirin may play a crucial role in the secondary prevention of both cardiovascular and cerebrovascular diseases [1,2,3]. Moreover, due to its direct, as well as indirect, damaging effect on gastric mucosa, the occurrence of aspirin-related peptic ulcers is increasing [4,5,6], and upper intestine bleeding still represents a major issue in patients chronically using aspirin.

The effect of aspirin has been assessed in the last forty years. In particular, it is known that aspirin inhibits cyclooxygenase-1 (COX-1) in platelets, leading to a reduction in platelet thromboxane A_2_ (TXA_2_) and the subsequent inhibition of platelet adhesion and aggregation, which are major steps that contribute to preventing cardiovascular complications [6,7]. However, the activity of aspirin is accompanied by a reduction in the protective activity exerted by the biosynthesis of gastric mucosal prostaglandin E_2_ (PGE_2_), an effect that increases the risk of gastric lesions and bleeding [8]. However, the inhibition of COX-1 as a result of taking aspirin is accompanied by the enhanced production of another class of COX-related products, namely, leukotrienes, mostly leukotriene B_4_, which leads to vasoconstriction and inflammation in gastric tissues, an effect associated with aspirin-induced gastric injury [9]. Finally, direct gastric mucosal lesion has been found to occur in patients undergoing long-term low-dose aspirin treatment, mostly due to its direct entry into gastric mucosal cells via non-ionic mechanisms, an effect associated with the back diffusion of hydrogen ions into mucosal cells [10].

The occurrence of damage of the gastric mucosa and the bleeding found in patients undergoing chronic oral treatment with low-dose aspirin have recently been confirmed in the ASCEND study [11], which elucidated that gastric injury counteracts the benefit derived from a more extended use of low-dose aspirin in the primary prevention in patients with diabetes, though further studies are required in this area. This, however, represents the basis for assessing therapeutic strategies aimed at combining gastro-protective drugs with aspirin in order to attenuate its potential damaging effect at the gastric level [11,12]. In fact, the use of protonic pump inhibitors (PPIs), as well as H_2_ histamine receptor antagonists, has been proven to be effective in protecting the gastric mucosa in patients undergoing low-dose aspirin treatment; this strategy, however, is associated with an increase in sanitary cost and is drug metabolism dependent [13,14]. Moreover, the use of aspirin formulations, in which the drug is combined with compounds that increase gastric pH, is associated with impaired aspirin absorption, as its entry into the cells is influenced by changes in its solubility, which decreases at higher pH levels [15,16,17]. Thus, the development of a better aspirin formulation still represents a challenge for researchers working in this area.

Recently, we developed and tested a new aspirin formulation based on an original process, which includes micronization and cogrinding with collagen of its crystalline form, thereby achieving a better drug absorption associated with significant protection of gastric mucosa [18].

In particular, both oral and sublingual administration of the novel aspirin formulation showed an early occurrence of serum concentration peak response compared to the standard crystalline drug formulation. This effect was associated with a decrease in both TXB_2_ serum concentration (the metabolite of platelet TXA_2_) and urinary 6-keto-PGF1α, which represents another reliable bio-marker of aspirin efficacy at the COX level [18].

Finally, experiments in rats have been performed in order to verify the attenuated impact of the novel aspirin formulation on the gastric mucosa. In particular, aspirin, either standard or collagen-cogrinded aspirin, at doses previously proven to produce gastric lesions, was given orally to rats to compare their damaging effects. Under these experimental conditions, we found that the severity of the aspirin-induced ulceration of the gastric mucosa was reduced when aspirin was cogrinded with collagen, an effect confirmed by histopathological studies [18]. Thus, the novel aspirin formulation seems to possess non-inferiority efficacy compared to standard aspirin and a better safety profile as evaluated in the experimental models of aspirin-related gastric damage.

The present randomized, double-blind, placebo-controlled study was performed in healthy volunteers in order to assess the safety profile of the novel formulation based on micronization and the cogrinding of aspirin with collagen.

## 2. Materials and Methods

### 2.1. Materials

Type I bovine collagen hydrolysate was purchased from LapiGelatin (Pistoia, Italy). Aspirin (acetylsalicylic acid), maltodextrin, microcrystalline cellulose, sodium carboxy-methyl starch, sucralose and magnesium stearate were purchased from Sigma-Aldrich (Milan, Italy).

### 2.2. Preparation of Aspirin Formulations

A mixture of hydrolyzed bovine collagen and crystalline aspirin was micronized and cogrinded in a ratio of 1 to 1 by means of a pin rotor mortar, which leads to micronization of powder particles at <40 µm (Pulverisette 14, Fisher, Idar Oberstein, Germany). Optical microscopy showed, after 20 min, the disappearance of micro-crystals of aspirin, replaced by amorphous particles surrounded by collagen. The amorphization of aspirin was confirmed by means of differential scanning calorimetry (DSC), performed with Perkin Elmer apparatus DSC7 and calibrated with Indium. The samples were examined with a scanning speed of 5.0 C/min. Granular aspirin was tested, highlighting the melting peak in the range of 133.9–136.8, which characterizes the crystalline building. In contrast, when evaluating aspirin micronized and cogrinded with bovine collagen hydrolysate via DSC, the melting peak between 135 °C and 138 °C disappeared. The aspirin amorphization after cogrinding with collagen was also confirmed by Raman microspectroscopy, as previously described [18].

In the second step, standard crystalline aspirin and aspirin micronized and cogrinded with collagen powders were used to obtain oral and sublingual formulations (tablets) by means of mechanical compression (Ronchi, Italy) under good manufacturing procedure (GMP-Institute of Research for Food Safety & Health, University of Catanzaro, Catanzaro, Italy) conditions. Microcrystalline cellulose, sodium carboxy-methyl starch, sucralose and magnesium stearate were thus used as excipients. When required, maltodextrin was used to obtain a homogeneous final weight for each tablet, as well as in the formulations for placebo. Placebo was obtained by substitution of aspirin and/or collagen in tablets with maltodextrin.

### 2.3. Study Design

A randomized, double-blind, placebo-controlled study was performed to evaluate the effect of oral, as well as sublingual, standard aspirin and micronized and collagen-cogrinded aspirin on gastric mucosal lesions and on serum TXB_2_ and 11-dehydro-TXB_2_ urinary levels. The study was carried out in 200 healthy volunteers, and data were compared with a placebo-treated group. The study complied with the principles of the Good Clinical Practice International Conference on Harmonization rules, was performed according to the CONSORT Statement and its checklist (http://www.consort-statement.org/, accessed on 9 January 2022; see Supplementary Materials) and was approved by the Regional Ethics Committee (extension study of EudraCT N. 2013-002980-24, 1 July 2013). Each study participant provided written informed consent. Inclusion and exclusion criteria are listed in the Supplementary Materials.

A total of 200 healthy volunteers were randomized using computerized random number generation by an independent investigator (CIRM, Milan, Italy) on a double-blind basis and randomly assigned to 10 groups (20 subjects each) according to the type and dose of aspirin or placebo (Table 1 summarizes subjects’ demographics). In particular, healthy volunteers received 7-day, once-daily treatment regimens: Groups A and B received oral and sublingual placebo, respectively; Groups C and D received standard oral aspirin of 100 mg or 50 mg, respectively; Groups E and F received sublingual aspirin of 100 mg or 50 mg, respectively; Groups G and H received oral micronized and collagen-cogrinded aspirin of 100 mg or 50 mg, respectively; and, finally, Groups I and L received sublingual micronized and collagen-cogrinded aspirin of 100 mg or 50 mg, respectively. On day 7, healthy volunteers entering the study received two low-fat meals (lunch and dinner at 12 and 18 h, respectively). Only mineral water was allowed.

Gastroscopy and blood and urine sample collection for analytical tests were performed in fasted volunteers on days 1 and 7 alongside the administration of the first and last doses of the drugs or the placebo. All of the procedures required the supervision of an investigator, who also instructed the healthy volunteers on the correct procedures for taking the aspirin or placebo at their home. A pill count was performed on day 7 in order to verify the compliance and adherence of subjects to the assigned treatment, which was 100%. This was also confirmed by contacting healthy volunteers by telephone on days 2–6. All subjects were instructed to not eat any meal for at least 6 h after taking the aspirin or placebo. Finally, drinking water was not allowed for at least 1 h before and after taking the aspirin or placebo.

Before administration of aspirin formulations or placebo and after the sixth dose, all subjects performed a 24 h urine collection in order to study the effect of aspirin on urinary levels of 11-dehydro-TXB_2_ (by means of ELISA immunoassay; BioRad, Milan, Italy).

Plasma TXB_2_ and urinary 11-dehydro-TXB_2_ were validated for precision and accuracy according to EMA guidelines. The mean serum levels of TXB_2_ and urinary 11-dehydro-TXB_2_ in healthy volunteers were 306 ± 45 ng/mL and 465 ± 58 pg/mg creatinine, respectively. Changes in these parameters were assumed to calculate the % response after aspirin treatment.

### 2.4. Endoscopy and Collection of Gastric Mucosal Samples for Histopathology

Gastroduodenal endoscopy was performed in healthy volunteers enrolled in the study after they fasted overnight, with an Olympus GIF-XQ 240 flexible gastroscope (Olympus Corporation, Tokyo, Japan). A blinded endoscopist collected the pictures and video sequences necessary for assessing the status of gastric mucosa during gastroscopy in both placebo- and aspirin-treated patients. The extent of gastric lesions was scored via the so-called modified Lanza score (MLS) system (Grade 0 = no erosion/hemorrhage, Grade 1 = 1–2 lesions of erosion and/or hemorrhage found in one area of the stomach, Grade 2 = 3–5 lesions of erosion and/or hemorrhage localized in one area of the stomach, Grade 3 = 6–9 lesions of erosion and/or hemorrhage detected in one area of the stomach or no more than 10 lesions in two areas in the stomach, Grade 4 = erosion and/or hemorrhage detected in three areas in the stomach or no less than 10 lesions in the whole stomach, and Grade 5 = gastric ulcer). The MLS was assessed as a means of three evaluations made by three independent endoscopists unaware of the group composition of the subjects undergoing gastroduodenal endoscopy. Gastric mucosa biopsy was collected from three different areas representative of the total gastric surface. Gastric specimens were stained with hematoxylin–eosin, and the sub-epithelial microvessels were measured according to their short-axis section. A mean of thirty vessels for each subject was taken as representative for assessing microvascular impairment in the subepithelial vessels.

### 2.5. Statistical Analysis

For the evaluation of data in our comparison between the groups, we used the statistical software SPSS version 22. The data were expressed as mean ± SD, and statistical comparisons were made by parametric tests (Student’s *t*-test or repeated measures analysis of variance, followed by the Student–Newman–Keuls test). The Shapiro–Wilk test was used to evaluate the distribution of the normality data. A probability value of *p* < 0.05 was considered to be statistically significant.

## 3. Results

### 3.1. Dissolution Studies

We investigated the in vitro dissolution of standard aspirin, as well as micronized collagen-cogrinded aspirin, in 50 and 100 mg tablets. The procedures defined by the USP/NF monograph dissolution procedure for aspirin tablets were followed for dissolution studies. In particular, the dissolution profiles of the products were defined in samples determined at times 1, 3, 6 and 15 min with a Q point at 30 min. A modified test procedure was also performed by using dissolution media prepared in accordance with the USP reagents section on the preparation of buffers at pH 1.2 and 6.8, respectively.

For calibration procedures, solutions contained aspirin at a concentration of 1 mg/mL under the same pH conditions as those of the dissolution buffer. Solutions of 1mg/mL were also used to obtain the isosbestic point for aspirin. Measurements were performed via a Cary Model 50 spectrophotometer (Agilent Technologies, Santa Clara, CA, USA), which were carried out after the collection and filtering of each sample. The procedures were performed according to GMP requirements.

At the two pH values, the aspirin tablet formulations were dissolved to an extent of 92.5 ± 2 and 99.2 ± 3%, respectively, at 15 min, while at the same time, the standard aspirin tablet was dissolved to an extent of 47.6 ± 3 and 82.8 ± 3%, at the two pH values, respectively. The dissolution studies carried out in vitro showed that the tablet containing aspirin micronized and cogrinded with collagen is characterized by a substantially faster dissolution compared to standard aspirin tablets at pH conditions ranging from 1.2 to 6.8 levels. Moreover, a pH-dependent dissolution capacity was found for both forms of aspirin, with lower dissolution rates detected at lower pH levels.

In all of the 200 subjects enrolled, the study was completed according to protocols between January 2018 and December 2019 at the IRC-FSH (Institute of Research for Food Safety & Health, University of Catanzaro, Catanzaro, Italy). There were no significant differences in the demographic or clinical characteristics (e.g., age and sex) at baseline among the different groups (Table 1). None of the subjects experienced any consistent adverse events associated with aspirin, such as gastrointestinal hemorrhage or major abdominal symptoms.

### 3.2. Gastric Mucosal Injury Induced by Standard Aspirin or Aspirin Micronized and Cogrinded with Collagen

**Figure 1:**
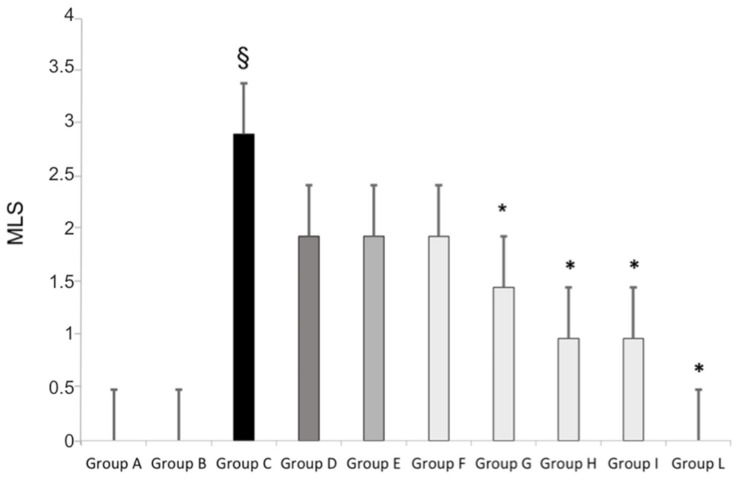
Gastric modified Lanza score (MLS) gastroscopy evaluation in healthy volunteers receiving placebo orally or sublingually (Groups A and B, respectively); receiving 100 or 50 mg of standard aspirin orally (Groups C and D, respectively); receiving 100 or 50 mg of standard aspirin sublingually (Groups E and F, respectively); receiving aspirin micronized and cogrinded with collagen orally (Groups G and H) or sublingually (Groups I and L). § *p* < 0.05 standard aspirin vs. placebo; * *p* < 0.05 collagen cogrinded aspirin vs. standard aspirin.

The changes in the MLS in each subject associated with the different treatments are shown in Figure 1. The median (range) MLS in Groups A and B (placebo) was 0 (0–1). A significant increase in the median MLS (3, 0) was observed in Group C, who received oral standard aspirin (100 mg daily for 7 days), in comparison with that in the groups receiving placebo (Groups A and B; Figure 1). This effect was moderately attenuated in Group D, who received 50 mg of oral standard aspirin, and in Groups E and F, who received standard aspirin sublingually at doses of 100 and 50 mg, respectively. The administration of oral aspirin micronized and cogrinded with collagen (Groups G and H), at doses of 100 and 50 mg, respectively, produced a further reduction in the aspirin impact on gastric mucosa. Indeed, the MLS was 2 and 1, respectively, compared to standard aspirin. A better response was found when micronized and collagen-cogrinded aspirin was given sublingually at doses of 100 mg (Group I—MLS 1) and 50 mg (Group L—MLS 0), the latter effect being similar to the one found with the placebo groups (Figure 1). Thus, aspirin micronized and cogrinded with collagen seems to display a better safety profile compared to standard aspirin.

### 3.3. Gastric Micro-Vessel Vasodilatation Induced by Standard as Well as Micronized and Collagen-Cogrinded Aspirin

**Figure 2:**
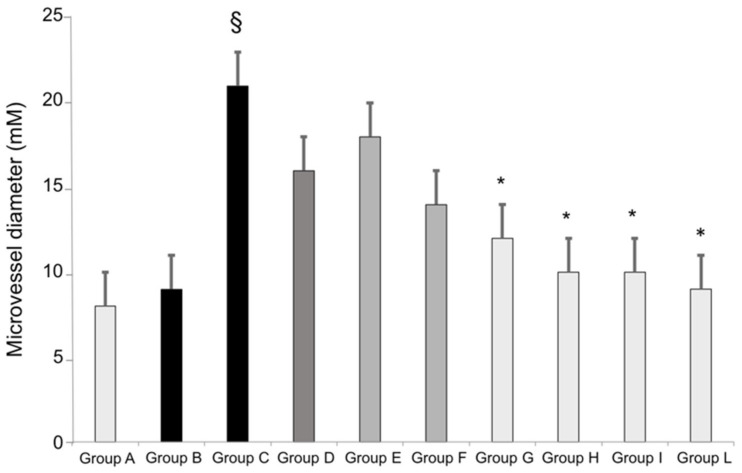
Gastric sub-epithelial micro-vessel diameter in healthy volunteers receiving placebo orally or sublingually (Groups A and B, respectively); receiving 100 or 50 mg of standard aspirin orally (Groups C and D, respectively); receiving 100 or 50 mg of standard aspirin sublingually (Groups E and F, respectively); receiving aspirin micronized and cogrinded with collagen orally (Groups G and H) or sublingually (Groups I and L). § *p* < 0.05 standard aspirin vs. placebo; * *p* < 0.05 collagen cogrinded aspirin vs. standard aspirin.

Treatment with oral standard aspirin (Groups C and D; 100 and 50 mg/daily, respectively) for 7 days significantly increased the median diameter of the sub-epithelial micro-vessels as compared with that of the groups receiving placebo (Groups A and B; Figure 2). A similar response was seen when standard aspirin, 100 and 50 mg/day, was given to healthy volunteers (Groups E and F, respectively; Figure 2). In contrast, this effect was found to be attenuated when micronized and collagen-cogrinded aspirin was given to the study population. In particular, in healthy volunteers taking aspirin micronized and cogrinded with collagen orally (Groups G and I) or sublingually (Groups H and L), a significant decrease in the diameter was found as compared with that of the standard aspirin regimen; in fact, the median diameter of the micro-vessels in the sublingual aspirin group was almost the same as the one found in the placebo group (Figure 2).

### 3.4. TXB_2_ and Urinary 11-Dehydro-TX B_2_ Determinations

The administration of oral aspirin, as well as sublingual aspirin (100 and 50 mg), either standard or micronized and cogrinded with collagen, was associated with decreased TXB_2_ serum levels as detected at day 7 compared to the groups receiving placebo. No significant changes were seen among the different regimens of aspirin treatment. Moreover, urinary measurements of 11-dehydro-TXB_2_ showed a similar response, thereby confirming that aspirin micronized and cogrinded with collagen showed a non-inferiority response to the COX enzyme compared to the crystalline standard formulation (Table 2).

The administration of standard aspirin and aspirin micronized and cogrinded with collagen given orally or sublingually did not produce any change in routine blood analytical biomarkers. In addition, no side effects or adverse drug reactions were described. Finally, the compliance and adherence were 100% in all groups and all the enrolled subjects in the study.

## 4. Discussion

The occurrence of gastric lesions in patients undergoing low-dose aspirin treatment for cardiovascular risk prevention represents a relevant issue, which, in some cases, limits the extensive use of such an antiplatelet drug [19,20].

In particular, data originating from very recent clinical trials and a meta-analysis performed on this topic confirmed that the efficacy of low-dose aspirin in the primary prevention of cardiovascular risk is seriously counteracted by the concomitant increased risk of gastric bleeding [21,22,23,24]. Thus, the limitation of aspirin-induced gastric injury and the development of a better aspirin may be considered relevant challenges for the research in this area [18,25,26].

The pathogenesis of aspirin-related gastric injury is complex and involves many players, including a reduced production of the protective gastric mucous associated with changes in gastric pH and direct gastrolesive action [27]. Furthermore, the dysregulation of nitric oxide (NO) production has been shown to contribute to the gastric lesions found in patients undergoing aspirin treatment. This fits with the evidence showing that aspirin induces inflammation in the gastric mucosa [28,29] as expressed by the dilatation of gastric microvessels.

Here, we reported that a better formulation of aspirin may represent an innovative way to maintain consistent antiplatelet activity as found with standard aspirin with a significant reduction in gastric injury and a better safety profile.

In particular, the micronization of aspirin and the cogrinding of the crystalline form of this drug with collagen lead to an innovative formulation that enhances drug absorption, thereby reducing gastric lesions associated with 7-day treatment with this drug. This confirms the previous evidence, in which we measured the serum concentration of aspirin both standard and micronized and cogrinded with collagen. These data are not surprising, as it is known that the micronization of crystalline drugs is clearly accompanied by an enhanced absorption of aspirin. In particular, evidence has been accumulated that micronization, which is able to reduce the size of crystalline aspirin, is associated with better drug absorption throughout both the gastric and sublingual mucosa. This has been confirmed by evidence obtained by means of Raman spectroscopy, which showed that the micronization of aspirin leads to a complete de-structuring of the crystalline form of the drug, an effect that has been demonstrated to increase the speed and rate of the absorption of many drugs, including aspirin. However, cogrinding aspirin with collagen leads to significant gastric protection. Indeed, collagen has been shown to produce both direct and indirect protection of the gastric mucosa, mostly due to its effect on gastric mucous. This effect occurs with no changes in gastric pH, compared to many of the compounds combined with aspirin in recent years, which are associated with an elevation of gastric pH. In particular, this effect is associated with a reduction in aspirin-related gastric injury, with the consequence, however, of poor aspirin absorption and subsequent reduced aspirin activity.

The innovative formulation of aspirin micronized and cogrinded with collagen seems to resolve many of the previous issues found with standard aspirin. Indeed, the extent of gastric erosions subsequent to oral aspirin was reduced when aspirin was micronized and cogrinded with collagen as detected via gastroscopy and measured via the Lanza’s score [30]. In particular, the use of the sublingual formulation of the micronized aspirin occurred with no changes in the gastric mucosa compared with standard aspirin. However, this effect was associated with comparable effects of novel aspirin formulation in the biomarkers of antiplatelet activity. Indeed, both the levels of TXB_2_ (the footprint of aspirin activity on platelet COX) and the levels of 11-dehydro-TXB_2_ (the urinary metabolite of TXA_2_) were comparable when using both sublingual and oral aspirin micronized and cogrinded with collagen when compared to standard aspirin, suggesting a non-inferiority profile of the novel formulations in the efficacy of aspirin. The better safety profile of novel aspirin is confirmed by the data from histopathological studies, which showed that the diameter of the microvessels of the gastric mucosa is reduced in subjects undergoing 7-day treatment with novel aspirin compared to the standard one, an effect that may be explained on the basis of the anti-inflammatory role of collagen when combined with aspirin, as expected with the micronized formulation compared to the standard, crystalline drug.

## 5. Conclusions

In conclusion, the present study demonstrated that low-dose standard aspirin induces gastric mucosal injury in healthy volunteers, an effect associated with the dilatation of the micro-vessels of gastric tissues. This response was attenuated when aspirin was micronized and cogrinded with collagen, with no significant changes in the ability of the new formulations to inhibit thromboxane formation.

Further studies are required in patients to verify the efficacy and safety of better aspirin formulations in both primary and secondary cardiovascular risk prevention in patients.

## Figures and Tables

**Table 1:** Demographics of healthy volunteers enrolled in the study. Values are expressed as mean ± SD.

Group	N.	Age (Years)	Gender (Male/Female)	Body Weight (Kg)	Body Mass Index (Kg/m^2^)	Smoking	Concomitant Treatment
A—placebo oral	20	32 ± 6	10 M and 10 F	68 ± 8	23 ± 2	0	0
B—placebo sublingual	20	35 ± 4	10 M and 10 F	66 ± 7	25 ± 4	0	0
C—oral standard aspirin 100 mg	20	34 ± 4	9 M and 11 F	66 ± 6	24 ± 4	0	0
D—oral standard aspirin 50 mg	20	34 ± 5	11 M and 9 F	68 ± 7	23 ± 5	0	0
E—sublingual standard aspirin 100 mg	20	34 ± 5	11 M and 9 F	65 ± 8	23 ± 3	0	0
F—sublingual standard aspirin 50 mg	20	35 ± 5	9 M and 11 F	68 ± 8	24 ± 3	0	0
G—oral micronized collagen-cogrinded aspirin 100 mg	20	33 ± 4	10 M and 10 F	67 ± 6	24 ± 4	0	0
H—oral micronized collagen-cogrinded aspirin 50 mg	20	34 ± 5	11 M and 9 F	66 ± 6	25 ± 3	0	0
I—sublingual micronized collagen-cogrinded aspirin 100 mg	20	35 ± 4	10 M and 10 F	66 ± 8	25 ± 4	0	0
L—sublingual micronized collagen-cogrinded aspirin 50 mg	20	34 ± 4	9 M and 11 F	68 ± 8	23 ± 5	0	0

**Table 2:** The effect of standard aspirin, collagen-cogrinded aspirin and placebo, given orally or sublingually to healthy volunteers, on serum TXB_2_ (ng/mL) and urinary 6-dehydro-TXB_2_ (pg/mg creatinine) at Time 0 before treatment and after 7 days of treatment. Values are expressed as mean *±* SD * *p* < 0.05 treatment vs. placebo.

Group	Serum TXB_2_ Time 0	Serum TXB_2_ 7 Days	Urinary 11-dehydro-TXB_2_ Time 0	Urinary 11-dehydro-TXB_2_ 7 Days
A—placebo oral	302 *±* 44	278 *±* 48	485 *±* 54	490 *±* 58
B—placebo sublingual	298 *±* 38	281 *±* 45	498 *±* 50	486 *±* 46
C—oral standard aspirin 100 mg	286 *±* 40	38 *±* 12 *	485 *±* 52	86 *±* 26 *
D—oral standard aspirin 50 mg	295 *±* 38	71 *±* 18 *	505 *±* 48	108 *±* 27 *
E—sublingual standard aspirin 100 mg	304 *±* 42	48 *±* 14 *	495 *±* 50	95 *±* 18 *
F—sublingual standard aspirin 50 mg	302 *±* 35	70 *±* 15 *	502 *±* 46	118 *±* 22 *
G—oral micronized collagen-cogrinded aspirin 100 mg	300 *±* 44	36 *±* 12 *	494 *±* 54	77 *±* 16 *
H—oral micronized collagen-cogrinded aspirin 50 mg	286 *±* 40	64 *±* 15 *	506 *±* 54	106 *±* 20 *
I—sublingual micronized collagen-cogrinded aspirin 100 mg	290 *±* 44	30 *±* 14 *	496 *±* 48	66 *±* 18 *
L—sublingual micronized collagen-cogrinded aspirin 50 mg	302 *±* 38	46 *±* 20 *	502 *±* 48	88 *±* 18 *

## Data Availability

The data presented in this study are available upon request from the corresponding author.

## Supplementary Materials

The following are available online at https://www.mdpi.com/article/10.3390/pharmaceutics14010187/s1, Clinical Study Protocol.
