# Supplementary material for: Efficacy and Safety of Novel Aspirin Formulations: A Randomized, Double-Blind, Placebo-Controlled Study
Source: Pharmaceutics. 2022 Jan 13;14(1):187. doi: 10.3390/pharmaceutics14010187 (PMC8779026; doi:10.3390/pharmaceutics14010187)
Supplement: Supplementary file 1 [file pharmaceutics-14-00187-s001.zip › pharmaceutics-1531448-supplementary.pdf]

pharmaceutics

Supplementary Materials: Efficacy and Safety of Novel Aspirin Formulations: A Randomized, Double-Blind, Placebo-Controlled Study

Rocco Mollace, Micaela Gliozzi, Roberta Macrì, Annamaria Tavernese, Vincenzo Musolino, Cristina Carresi, Jessica Maiuolo, Carolina Muscoli, Carlo Tomino, Giuseppe M. Rosano, Massimo Fini, Maurizio Volterrani, Bruno Silvestrini and Vincenzo Mollace

CLINICAL STUDY PROTOCOL

Study Title:

"Efficacy and safety of novel aspirin formulation: a randomized, double blind, placebo-controlled study ".

Study Drug:

Standard acetylsalicylic acid in oral and sublingual (50 and100 mg) formulations and micronized, collagen co-grinded acetylsalicylic acid in oral and sublingual (50 and 100 mg) formulations

Indication Primary:

Prevention of cardiovascular disease

Development Phase:

Phase IIb

Protocol Code:

ASA-001

Promotore:

No Profit

Investigator:

Vincenzo Mollace

Executive Scientific Committee:

Vincenzo Mollace, Bruno Silvestrini, Fabrizio Stocchi, Cristiana Vitale

EudraCT Number:

2013-002980-24

Version and Date:

Final
July 1st 2013

INDEX

1. BACKGROUND

2. STUDY OBJECTIVES AND PURPOSE

3. STUDY DESIGN

3.1. Primary objectives

3.1.a. Efficacy

3.1.b. Safety

3.2. Secondary objectives

3.2.a. Efficacy

4. DESIGN and METHODOLOGY of the planned research

4.1. Experimental design

4.1.a. Study plan and investigational schedule

5. SUBJECTS

5.1. Inclusion criteria

5.2. Non-inclusion criteria

5.3. Sample size calculation

6. METHODS OF ASSESMENT

a. Assessment of blood pressure
b. Assessment of cardiovascular risk with the Systematic Coronary Risk Evaluation (SCORE)chart
c. Assessment of faecal occult blood
d. Laboratory analyses
e. Assessment of platelet aggregation
f. Urea breath test (UBT)

7. MEASURES TO MINIMIZE BIAS

8. STUDY PRODUCT AND BLINDING SYSTEMS

8.1. Products administered

8.1.a. Description of the study products8.1.a1 Pharmaceutical form

8.1.a2 Unit dosage 8.1.a3 Composition

8.2. Treatment management

8.3. Management of blinding systems

8.4. Discontinuation of the study

8.4.a. Premature discontinuation of the study

9. SOURCE DATA

10. WITHDRAWAL OF PATIENTS

10.1. Patient withdrawal and discontinuation of study medication

10.1.a. Withdrawal criteria

10.1.a2. Procedure

11. TREATMENT OF PARTICIPANTS

11.1. Treatments administered

11.2. Treatments dispensing

11.3. Previous and concomitant treatments

11.3.1. Previous treatments

11.3.2. New concomitant treatments during the study

11.4. Treatment compliance

11.5. Arrangements after the discontinuation of the study treatment

12. ASSESSMENT OF EFFICACY

12.1. Efficacy measurements

13. ASSESSMENT OF SAFETY

13.1. Safety measurements

14. SECONDARY OBJECTIVES

14.1. Efficacy

15. POTENTIAL SIDE EFFECTS, RISKS or HAZARDS for research participants

15.1. Responsibilities of investigator

15.1.a. Recording of Adverse Events in the Case Report Form15.1.a1. Events to be recorded

15.2. Recording methods

15.3. Follow-up of Adverse Events

15.4. Procedure for an Event requiring immediate notification

15.5. Evaluation of causality

15.6. Responsibilities of PI

16. STATISTICS

16.1. Responsibility for Analysis

16.2. Hypotheses/Estimation

16.3. Analysis Endpoints

16.3.1. Efficacy Endpoints

16.3.2. Primary Endpoint

16.3.3. Secondary Endpoints

16.3.4. Safety Endpoints

16.4. Analysis Populations

16.4.1. Efficacy Analysis Populations

16.4.2. Safety Analysis Populations

16.5. Efficacy Analysis

16.5.1. Primary Endpoint Analysis

16.5.2. Secondary Endpoint Analysis

16.6. Evaluation of Safety

16.7. Summaries of Baseline Characteristics

16.8. Interim Analysis

16.9. Determination of Sample Size

17. DIRECT ACCESS TO SOURCE DATA/DOCUMENTS

18. QUALITY CONTROL AND QUALITY ASSURANCE

18.1. Study monitoring

18.1.a. Before the study

18.1.b. During the study

18.2. Audit – Inspection

19. ETHICS

19.1. Ethics Committee(s)

19.1. Study conduct

19.1. Patient Information and Informed Consent

20. DATA HANDLING AND RECORD KEEPING

20.1. Study data

20.2. Data management

20.3. Archiving

21. INSURANCE

22. OWNERSHIP OF THE RESULTS – PUBLICATION POLICY

23. INTELLECTUAL PROPERTY

24. SUBSTANTIAL PROTOCOL AMENDMENT

25. FINAL STUDY REPORT

26. ORGANISATION OF THE CENTRE

27. REFERENCES

Appendix 1

INCLUSION AND EXCLUSION CRITERIA

4. SUBJECTS

Patients were eligible for participation in the present study if fulfilling the following criteria:

4.1. Inclusion criteria

- Men or women
- Age ≥18 years
- Healthy volunteers with no cardiovascular risk, assessed by using the SCORE system (<1% for 10-year risk of fatal CVD), according to the European Guidelines on cardiovascular disease prevention in clinical practice
- Written informed consent.

All enrolled **women** of reproductive age will undergo a pregnancy test and had to use birth control measures during the whole study.

All concomitant treatment(s) should have been initiated for at least 3 months and are expected to bemaintained at a fixed dose during the entire duration of the study.

4.2. Non-inclusion criteria

- Age <18 years
- Positive pregnancy test (βGCH) performed at the selection visit or results not available, women who are pregnant, women who are breast-feeding, women of childbearing potential not using estro-progestative or progestative or intrauterine contraception, or women using estro-progestative or progestative or intrauterine contraception, but who consider stopping it during the planned duration of the study
- Patients at very high or low cardiovascular risk, having a calculated SCORE ≥10%
- History of atrial fibrillation or atrial flutter
- Patient in treatment with or with indication to oral anticoagulants or other antithrombotic drugs
- Patient with history of hypersensitivity or intolerance with acetylsalicylic acid or other non-steroidal ant-inflammatory drugs (NSAIDs)
- Patients with any history of hereditary angioedema, idiopathic or associated with acetylsalicylicacid
- Patients with history of peptic ulcers or gastritis or history of gastrointestinal bleedingassociated to acetylsalicylic acid or other non-steroidal anti-inflammatory drugs (NSAIDs)
- Patients with severe gastro-intestinal tract disorders with possible influence on drug absorptionor electrolytes
- Patient with asthma or NSAID-precipitated bronchospasm
- Patients with known coagulation disorders or with abnormal platelet count (<100 000 per mm3or >400 000 per mm3) or with other acquired causes of impaired platelet aggregation, including uremia and paraproteinemia (monoclonal gammopathy)
- Patients with haemophilia or other bleeding disorders
- Patients with myeloproliferative disorders
- Patients with severe chronic kidney disease (GFR <30 mL/min/1.73 m2), using Cockcroft's formula:
- men = (140 - age) x weight (kg)/(0.814 x creatinine level (μmol/L))
- women = 0.85 x [(140 - age) x weight (kg)/(0.814 x creatinine level (μmol/L))]

- Patients with known liver disease or biliary obstructive disorders, with or without complications (chronic hepatitis, cirrhosis, etc) or with ALT or AST upper than 3 times the upper limit ofnormal laboratory range
- Patient with hyperkalemia
- History of alcoholism or drug abuse
- Patients unlikely to co-operate in the study or to comply well with treatment or with the studyvisits
- Participation in another study at the same time or within the preceding 30 days, (or a longerperiod in accordance to the local regulations)
- History of severe disease likely to interfere with the conduct of the study, severe uncontrolledinfection, evolving neoplasm
- History of severe mental or psychiatric disorder, severe depression or history of severedepression, e.g. requiring an hospitalization or at high risk of suicide attempt
- Endocrine diseases: uncontrolled dys-thyroidia, Cushing's syndrome, acromegalia, hyperparathyroidia
- Patient with a life expectancy of less than the 15 months duration of the study in opinion to theinvestigator
- Patients with HIV or taking drugs for HIV.

Concerning concomitant medications

The use of the following treatments prevents the patient from being selected: antidepressant such as citalopram, duloxetine, escitalopram, fluoxetine, fluvoxamine, paroxetine, sertraline or venlafaxine; other non-steroidal ant inflammatory drugs (NSAIDs) or another salicylate such as choline salicylate and/or magnesium salicylate or salsalate (Disalcid), ibuprofen, indomethacin, naproxen, and tiaprofenicacid when taken regularly, Adefovir, chlorpheniramine/ibuprofen/pseudoephedrine, iphenhydramine/ibuprofen, brinzolamide and dorzolamide ophthalmic, emtricitabine, immunoglobulin, dichlorphenamide, acetozolamide, sodium biphosphate/sodium phosphate, methazolamide, tacrolimus, influenza virus vaccine, sirolimus, hydrocodone, methotrexate, tenofovir, aminophylline, antihistamines, oral anticoagulants or other antithrombotic drugs, statins, nitrates, corticosteroids, cephalosporins, agents that cause urinary alkalinization (ie potassium or sodium citrate), antacids containing either aluminum and magnesium hydroxide or calcium carbonate two hours before acetylsalicylic acid dosing, dietary supplements (ginkgo, vitamin E, garlic, ginger, ginseng, omega-3 fatty acids (fish oil), local anaesthetics (procaine).
